# Impact of real-time fMRI working memory feedback training on the interactions between three core brain networks

**DOI:** 10.3389/fnbeh.2015.00244

**Published:** 2015-09-04

**Authors:** Qiushi Zhang, Gaoyan Zhang, Li Yao, Xiaojie Zhao

**Affiliations:** ^1^College of Information Science and Technology, Beijing Normal UniversityBeijing, China; ^2^School of Computer Science and Technology, Tianjin UniversityTianjin, China; ^3^State Key Laboratory of Cognitive Neuroscience and Learning, Beijing Normal UniversityBeijing, China

**Keywords:** large-scale brain network, working memory training, real-time fMRI, functional connectivity, insula

## Abstract

Working memory (WM) refers to the temporary holding and manipulation of information during the performance of a range of cognitive tasks, and WM training is a promising method for improving an individual’s cognitive functions. Our previous work demonstrated that WM performance can be improved through self-regulation of dorsal lateral prefrontal cortex (PFC) activation using real-time functional magnetic resonance imaging (rtfMRI), which enables individuals to control local brain activities volitionally according to the neurofeedback. Furthermore, research concerning large-scale brain networks has demonstrated that WM training requires the engagement of several networks, including the central executive network (CEN), the default mode network (DMN) and the salience network (SN), and functional connectivity within the CEN and DMN can be changed by WM training. Although a switching role of the SN between the CEN and DMN has been demonstrated, it remains unclear whether WM training can affect the interactions between the three networks and whether a similar mechanism also exists during the training process. In this study, we investigated the dynamic functional connectivity between the three networks during the rtfMRI feedback training using independent component analysis (ICA) and correlation analysis. The results indicated that functional connectivity within and between the three networks were significantly enhanced by feedback training, and most of the changes were associated with the insula and correlated with behavioral improvements. These findings suggest that the insula plays a critical role in the reorganization of functional connectivity among the three networks induced by rtfMRI training and in WM performance, thus providing new insights into the mechanisms of high-level functions and the clinical treatment of related functional impairments.

## Introduction

Research on large-scale brain networks in cognition have achieved great progress since this paradigm was proposed. The concept of large-scale brain networks emphasizes the conjoint function of brain areas working together in contrast to conventional simplistic mapping of cognitive constructs onto individual brain areas, and it is critical for gaining a deeper insight into the neural basis of cognition (Bressler and Menon, 2010). Among these functional brain networks, the central executive network (CEN; Petrides, 2005; Koechlin and Summerfield, 2007), the default-mode network (DMN; Raichle et al., 2001; Greicius et al., 2003; Zhu et al., 2013), and the salience network (SN; Downar et al., 2002; Critchley et al., 2004), which are known to be involved in working memory (WM), are considered the three core neurocognitive networks due to their critical roles in high-level cognition (Uddin et al., 2011). Interest in their interactions and the corresponding impact on brain functions has increased recently. Chen et al. demonstrated the causal neural mechanism by which the DMN was negatively regulated by the CEN and SN (Chen et al., 2013) while more researchers have emphasized the SN or the control of the insula over the CEN and DMN; for example, Sridharan found a switching mechanism between the SN and the other two networks (Sridharan et al., 2008). How these networks and this mechanism reconfigure and mature with development have been explored by many studies (Fair et al., 2007; Menon, 2013), and dysfunction of this mechanism has been found to be associated with psychiatric disorders, such as schizophrenia and autism (Moran et al., 2013; Uddin et al., 2014). Based on previous findings, Menon proposed a triple network model that helped synthesize them into a common framework for understanding dysfunction in these core neurocognitive networks across multiple disorders (Menon, 2011).

In the triple network model, the most significant node is considered to be the right fronto-insular cortex (rFIC) of the SN, and it acts as an integral hub in mediating dynamic interactions between other large-scale brain networks involved in externally oriented attention (the CEN) and internally oriented self-related mental processes (the DMN; Sridharan et al., 2008). This mechanism has been extended into research on neurological and psychiatric disorders because dysfunction in one core network can often impact the other two (Menon, 2011). Several diseases have been found accompanied by aberrant functional connectivity of the insula, such as schizophrenia (Moran et al., 2013), Alzheimer’s disease (Balthazar et al., 2014), drug addiction (Sutherland et al., 2012), and so on. Relatively fewer studies concerned this switching mechanism and its functions in normal individuals.

Real-time functional magnetic resonance imaging (rtfMRI) allows for the measurement and feedback of localized dynamic brain activities in individuals to train them to control their brain activities in specific brain areas (Ruiz et al., 2014). Recent studies in this field have focused mainly on the functional improvement of cognition and brain plasticity through feedback training (Niv, 2013; Sulzer et al., 2013). Volitional control over a number of brain areas has been achieved, including the control of the performance of motor skills through the motor-related cortex (deCharms et al., 2004; Beckmann et al., 2005; Yoo et al., 2008; Sitaram et al., 2012; Zhao et al., 2013), the control of WM through the left dorsal lateral prefrontal cortex (DLPFC; Zhang et al., 2013), the control of emotion through the right anterior insular cortex (Caria et al., 2010), and the control of visual sensitivity for detecting targets through the right inferior frontal gyrus (Rota et al., 2009, 2011). Further studies found that the volitional control of one single region may affect its connectivity with other regions. In Sergio’s experiment, schizophrenia patients managed to acquire self-control of the insular cortex, which led to behavioral modifications and an enhancement in the neural connectivity of brain networks (Ruiz et al., 2014). Furthermore, the up-regulation of the premotor cortex, which is engaged in motor execution (ME) and imagery (MI) tasks, induced alterations in the functional connectivity of both the ME-related and MI-related motor networks (Hui et al., 2014). To date, these investigations have focused on the impact of rtfMRI on single networks, and few studies involved large-scale networks, which contribute to the training process and performance improvements.

WM refers to the temporary holding and manipulation of information during the performance of a range of cognitive tasks (Baddeley, 1992), and WM training is promising for improving an individual’s cognitive functions (Klingberg, 2010). Behavioral training studies have demonstrated that several networks, such as the CEN and DMN, can be modulated by WM training. Jolles found that functional connectivity of young adults between the right middle frontal gyrus (MFG) and other regions of the fronto-parietal network increased after WM training (Jolles et al., 2013). Takeuchi found that functional connectivity between the medial PFC (MPFC) and precuneus, which are key nodes of the DMN, also increased after WM training (Takeuchi et al., 2013). Furthermore, the strength of functional connectivity in the CEN and DMN both correlated with behavioral performance (Chen et al., 2009; Esposito et al., 2009) and are both modulated by memory load (Newton et al., 2011). However, how multiple networks interact during training and how training affects their interactions is unclear. With rtfMRI being applied to neuroimaging studies, our recent research successfully trained subjects to gain control over the activation of the left DLPFC (lDLPFC) through rtfMRI and proved that WM performance can be improved through self-regulation of activation in associated brain regions (Zhang et al., 2013). Although previous studies have provided preliminary evidence that feedback training may induce alterations in relevant brain networks that are correlated with behavioral performance (Ruiz et al., 2011), whether rtfMRI-based WM training affects the interactions between large-scale networks and if so, how this is done remains unclear.

In this paper, we explored the activity of the three core networks during rtfMRI-based WM training and examined the effects of training on the interactions between them. Based on our previous work (Zhang et al., 2013), we performed independent component analysis (ICA) to extract the CEN, DMN and SN that were engaged in the training. Then, we analyzed the dynamic changes in the functional connectivities between key nodes in the three networks and their relationships with behavioral performance. Based on the conclusions of previous studies that functional connectivities within the three networks can be altered and are associated with behavioral performance (Chen et al., 2009; Esposito et al., 2009) and a study that indicated that the insula acts as an integral Hub in mediating dynamic interactions between the CEN and DMN (Sridharan et al., 2008), we hypothesized that the functional connectivity within and between the three networks may be altered by rtfMRI-based WM training. Moreover, these changes, especially those associated with the insula, may be related to the improvements of behavioral performance.

## Materials and Methods

### Participants

The same dataset as that in one of our previously published articles was used here (Zhang et al., 2013). A total of thirty healthy, right-handed subjects participated in this experiment. All subjects were randomly assigned to the experimental group (8 males and 7 females, mean age: 21.47 ± 3.83 years) or the control group (8 males and 7 females, mean age: 21.87 ± 3.41 years). None of the participants had any history of psychiatric or neurological disorders or had experience in memory training or instrumental learning.

All participants provided written consent before the experiment. The experiment was approved by the Institutional Review Board (IRB) of the State Key Laboratory of Cognitive Neuroscience and Learning in Beijing Normal University.

### rtfMRI Data Acquisition

MRI scanning was performed on a SIEMENS 3.0 T scanner at the MRI center of Beijing Normal University. Functional brain images were acquired with a single-shot T2*-weighed echo-planar imaging (EPI) sequence (TE = 30 ms, TR = 2000 ms, flip angle = 90°, In-plane resolution = 3.125 × 3.125 mm^2^, matrix size = 64 × 64, slice = 33; slice thickness = 3.60 mm). High-resolution anatomic images were acquired with a T1-weighted magnetization-prepared rapid gradient echo (MPRAGE) sequence (matrix size = 256 × 256, 176 partitions, 1 mm^3^ isotropic voxels, TR = 2530 ms, TE = 3.45 ms, flip angle = 7°). To reduce movement, two foam cushions were used to immobilize the subjects’ heads.

### Experimental Procedure

The entire experimental procedure included two rtfMRI training sessions separated by 7 days. Each session started with a 10 min T1-weighted scan, then every subject completed a region of interest (ROI) localizer run composed of interleaved four digital 0-back blocks and three digital 3-back blocks, which helped the experimenters select the subject’s target ROI in the lDLPFC. Following the localizer run were four feedback runs. Each feedback run consisted of four 60 s task blocks and five 30 s baseline blocks. A graduated thermometer was presented on the screen and was updated once per TR during the training. It provided visualization of the subject’s brain activation in lDLPFC and served as feedback for the participants. Subjects in the experimental group were required to recite backward the sequences they provisionally defined to increase the number of bars, namely, activities of the target ROI, as high as possible. Trying different complexity or reciting speed was suggested as possible strategies. Subjects in the control group completed the same experimental procedure and received the same instructions, except that the feedback information they received were randomly chosen from an experimental participant who showed an intermediate level of self-regulation effect. All the subjects attended behavioral tests before and after each training session, including a forward and backward digit span task, and a letter memory task. The forward and backward digit span task measured the total maximum length of a digit sequence that a subject could recite forwardly and backwardly. The letter memory task involved 10 lists of letter sequence with varied length from 6–15 letters; when a list ended, the subjects were asked to enter the last 4 letters using the keyboard within a time limit of 6 s. More details about the experimental procedure can be found in the report of our previous study (Zhang et al., 2013).

### Offline Data Analysis

#### Data Preprocessing

The fMRI data were preprocessed using SPM8 software^1^ after the first four volumes were discarded to eliminate T1 equilibration effects. The steps included slice timing, head motion correction, normalization to the Montreal Neurological Institute (MNI) space, reslicing into a resolution of 3 × 3 × 4 mm^3^ and spatial smoothing using a Gaussian kernel with full-width at half maximum (FWHM) of 8 mm.

#### Independent Component Analysis

We performed ICA on the rtfMRI data of all feedback runs of all subjects in the experimental group and the control group to identify spatially independent networks engaged in our WM training and to measure the training effects on these networks. ICA was carried out using Group ICA Toolbox (GIFT v2.0h^2^). Based on the minimum description length (MDL) criteria, the optimal number of independent components (ICs) was constrained to 22. Principle component analysis (PCA) was used to perform two data reduction steps. The reduced data were then decomposed using the Infomax ICA algorithm into a series of spatially independent maps and their corresponding time courses, which were back-reconstructed subsequently for each subject to produce individual spatial maps and time courses. The CEN, DMN and SN were selected according to their spatial patterns described in previous studies (Raichle et al., 2001; Uddin et al., 2011; Xue et al., 2012).

For each selected IC, a two-way repeated-measure ANOVA, with run as the within-subjects factor and group as the between-subjects factor, was conducted in SPM8 and corrected using false discovery rate (FDR) at an overall alpha level of 0.001. Based on the ANOVA analysis at the group level, the ROIs of the three networks were defined as a spherical region centered on the local maximum peak with a radius of 6 mm.

#### Functional Connectivity Analysis

The mean time course of each ROI in each training run for every subject was extracted, and the Pearson correlation coefficients between each ROI pair within and between the three networks were calculated and transformed into *z*-scores using Fisher’s *r*-to-*z* transformation. For subjects in each group, the *z*-scores of ROI pairs were entered into SPSS 16.0 for a one-way repeated-measure ANOVA with the training run as the main factor (eight levels). The significance of the multiple comparisons was corrected using FDR at the level of 0.05.

For those ROI pairs with significant main effect (*p* < 0.05), we calculated the correlation coefficients between the changes in functional connectivity from the last training run (run 2D) to the first training run (run 1A) and the behavioral changes from post-test to pre-test.

## Results

### Independent Component Analysis and ROI Selection

**Figure 1:**
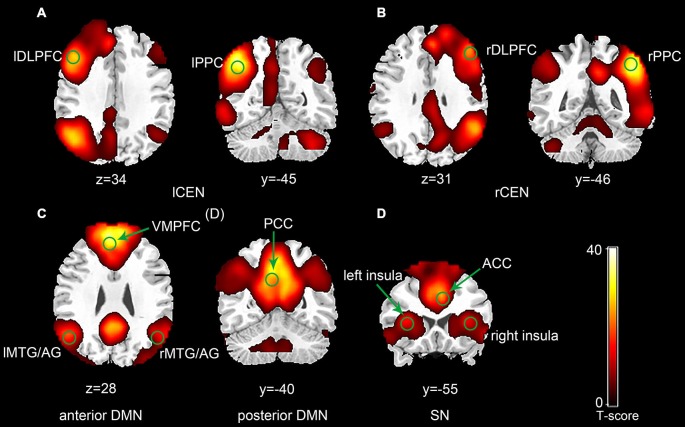
**Spatial maps of selected three network components (*p* < 0.001, false discovery rate (FDR) correction; Left side is on the reader’s left).** The central executive network (CEN) was typically split into a left component **(A)** and a right component **(B)**. The default mode network (DMN) was split into an anterior component **(C)** and a posterior component **(D). (E)** Represents the salience network (SN).

The ICA generated two components for CEN (left and right), two components for DMN (anterior and posterior) and one component for SN (Figure 1). Each of the components was identified by comparing their spatial patterns with those described in previous studies after visual inspection to discard noise components (Raichle et al., 2001; Uddin et al., 2011; Xue et al., 2012). The CEN consisted of brain areas, such as the bilateral DLPFC and posterior parietal cortex (PPC). The DMN was anchored in the ventral medial prefrontal cortex (VMPFC), posterior cingulate cortex (PCC), bilateral middle temporal gyrus (MTG) and angular gyrus (AG). Key brain areas in the SN included the anterior cingulate cortex (ACC) (Esposito et al., 2009) and bilateral insula. No significant interactions between run and group or significant main effect of run or group was observed in any of the three networks by ANOVA in SPM8. Pair-wise comparison of run 2D with run 1A showed no significant difference in any of the three networks in each group (*p* > 0.05). The between-group comparison showed no difference in any of the three networks in run 1A or run 2D (*p* > 0.05). The ROI selection were constrained to the eleven key brain areas in the three networks mentioned above, including the bilateral DLPFC, bilateral PPC, bilateral insula, ACC, VMPFC, PCC and bilateral MTG/AG (Table 1).

**Table 1:** **Coordinates of the ROIs in the CEN, DMN and SN**.

Network	Region	L/R	*x*	*y*	*z*	BA	*T*_max_
CEN	DLPFC	L	−42	26	38	9	30.5
		R	45	23	38	9	28.79
	PPC	L	−45	−52	46	40	33.95
		R	42	−52	50	40	35.19
DMN	VMPFC	L/R	−3	47	−10	11	25.8
	PCC	L/R	−3	−55	22	31	23.45
	MTG/AG	L	−45	−61	26	39	18.77
		R	51	−64	26	39	12.69
SN	ACC	L/R	6	23	26	24	17.61
	Insula	L	−33	20	6	13	15.35
		R	36	20	6	13	13.57

### Functional Connectivity Changes Within and Between the Three Networks

**Figure 2:**
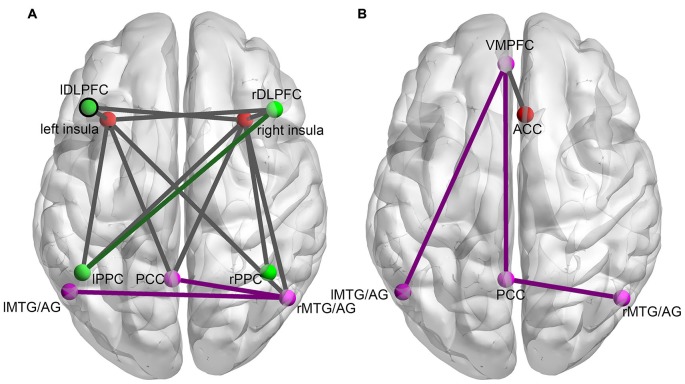
**The changes in functional connectivity between ROI pairs within and between the three networks induced by the rtfMRI training for the experimental group (A) and the control group (B).** Nodes of the same color represent ROIs in the same network (red nodes: SN; green nodes: CEN; purple nodes: DMN). Purple lines (connections within the DMN), green lines (connections within the CEN) and dark gray lines (connections between different networks) represent the functional connectivity with significant increases from run 2D to run 1A (FDR correction, *p* < 0.05).

The ANOVA analysis on the functional connectivity of all possible ROI pairs within and between the three networks revealed nineteen ROI pairs with a main effect in their functional connectivity in the experimental group and five ROI pairs with a main effect in their functional connectivity in the control group. The subsequent simple effect test on these functional connectivities showed that in the experimental group, the functional connectivity of fourteen ROI pairs increased significantly in the last training run (run 2D) compared to the first training run (run 1A; paired *t*-test, *p* < 0.05; Figure 2A). In the control group, only four ROI pairs showed a significant increase in functional connectivity from the run 1A to the run 2D (*p* < 0.05; Figure 2B). No significant difference in functional connectivity between run 1A and run 2D was found in the other five ROI pairs in the experimental group and the other one ROI pair in the control group.

### Correlation Between Functional Connectivity and Behavioral Performance

**Figure 3:**
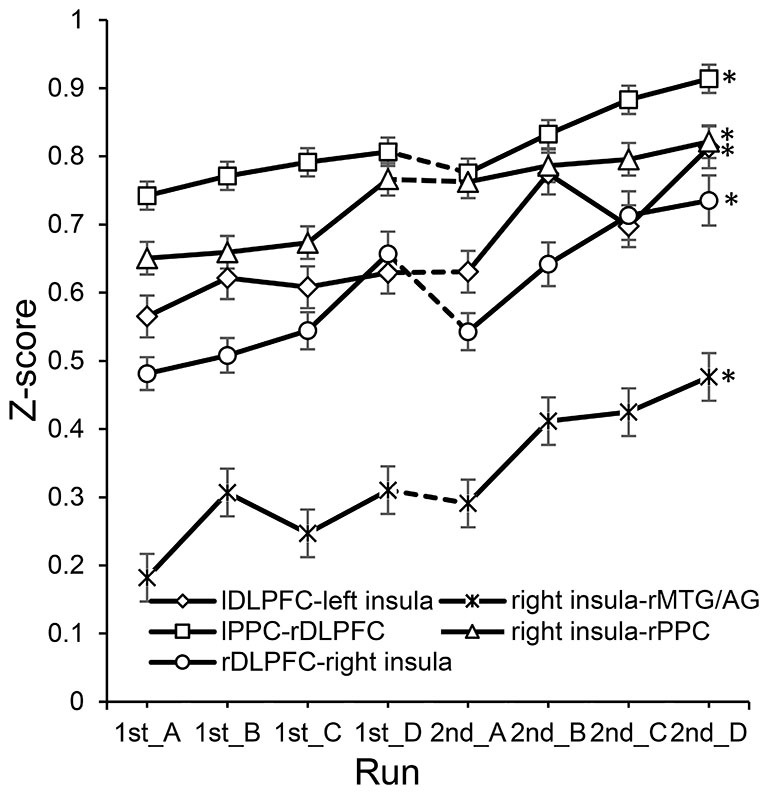
**The changes of functional connectivity correlated with performance in the experimental group**. *Represents significant difference between run 2D and run 1A (*p* < 0.05). Dotted lines between 1st_D and 2nd_A represent the 7 day break between two sessions.

In the experimental group, significant correlations between the increased functional connectivity from run 1A to run 2D and improved performance were mainly found between the SN and the other two networks (Table 2). These functional connectivity curves, which varied with the training progress, are illustrated in Figure 3. An overall progressive increase in *z*-scores was found by the linear regression analysis of the functional connectivity between the right insula and right MTG/AG (rMTG/AG) (*y* = 0.0372*x* + 0.1639, *R*^2^ = 0.8506, *p* = 0.001), the lDLPFC and left insula (*y* = 0.0312*x* + 0.5272, *R*^2^ = 0.7766, *p* = 0.004), the right PPC (rPPC) and right insula (*y* = 0.0263*x* + 0.621, *R*^2^ = 0.9104, *p* = 0.0002), the right DLPFC (rDLPFC) and right insula (*y* = 0.0355*x* + 0.4432, *R*^2^ = 0.8155, *p* = 0.002), and the left PPC (lPPC) and rDLPFC (*y* = 0.022*x* + 0.7155, *R*^2^ = 0.8488, *p* = 0.001). No significant correlations between the changes in functional connectivity of any ROI pairs and improvements in behavioral performance were detected in the control group.

**Table 2:** **The correlation of the increased functional connectivity with the behavioral improvements**.

	Letter memory			Digit span		
Network	ROI pair	*r*	*p*	ROI pair	*r*	*p*
SN-DMN	Right insula-rMTG/AG	0.472	0.038	Right insula-rMTG/AG	−0.706	0.002
SN-CEN	lDLPFC-left insula	0.475	0.004	lDLPFC-left insula	−0.501	0.003
	rPPC-right insula	0.585	0.011
	rDLPFC-right insula	0.499	0.029
CEN-CEN	lPPC-rDLPFC	0.472	0.037

## Discussion

The present study investigated the interactions induced by the self-regulation of lDLPFC among the three core networks, including the CEN, DMN and SN, in a WM feedback training experiment using rtfMRI. Our results showed that the functional connectivity between the SN and CEN, between the SN and DMN, and within the CEN changed significantly as the training progressed. Moreover, some of these connectivities were found to be correlated with behavioral improvements. Notably, the majority of these connectivities were linked with the right insula. These findings suggest that the right insula may play a critical role in modulating the functional connectivities within the triple network model during rtfMRI feedback training, which may lead to the improvements of behavioral performance.

Previous studies indicated that the CEN, DMN and SN were three core networks in WM tasks (Menon, 2011). The CEN is generally considered to be responsible for the information maintenance and manipulation in WM as well as decision making in the context of goal directed behavior (Sridharan et al., 2008). The DMN is typically deactivated during most stimulus-driven cognitive tasks (Raichle et al., 2001; Greicius et al., 2003), and alterations in the activity and connectivity of brain regions within the DMN may be associated with different WM performances (Chen et al., 2009; Esposito et al., 2009; Pyka et al., 2009). DMN dysfunction has been found in several disorders that alter episodic memory, autobiographical memory and self-related mental processes (Menon, 2011). The SN, especially the insula, plays an important role in saliency detection, attentional capture enhanced by error signals and dynamic cognitive control in the triple network model (Menon, 2011), and it can facilitate access to attention and WM resources when a salient event is detected (Menon and Uddin, 2010). In agreement with the results of these studies, our research demonstrated that rtfMRI-based WM training also triggered the activities of the three networks (Figure 1). However, the training effects based on rtfMRI were reflected mainly in the changes of functional connectivity within and between the three networks rather than the changes of the three networks themselves.

Changed functional connectivity within networks was only found in the CEN and DMN. According to a previous publication, WM maintenance requires the coordination of multiple brain regions, including the PFC, the parietal cortex, and posterior unimodal association areas (Gazzaley et al., 2004). Moreover, another study indicated that individuals with autism had lower functional connectivity between the left parietal and right frontal regions than normal controls in an N-back WM task with letters. The author attributed this difference to the different level of cognitive strategies the two groups adopted. In other words, a relatively easier cognitive strategy may lead to lower functional connectivity between the left parietal and right frontal regions (Koshino et al., 2005). The post-experiment questionnaires in our study indicated that most subjects managed to enhance the activation of the target ROIs by increasing the difficulty and randomness of the generated sequences (Zhang et al., 2013). Thus, the change in functional connectivity between the lPPC and rDLPFC may be the result of the subjects’ memory strategy adjustments. Unlike the CEN, the specific functions of individual brain regions in the DMN may be quite different, but as a whole, this network has been reported to be in charge of self-monitoring, planning, problem solving, internal monitoring and episodic memory encoding (Gusnard et al., 2001; Pyka et al., 2009; Spreng et al., 2009). Analysis of intrinsic connectivity within the DMN during an N-back task found that the degree of intrinsic correlation within the network increased with task load (Newton et al., 2011). Therefore, functional connectivity changes in the DMN observed in both the experimental and control group may be interpreted as the result of the DMN’s self-evaluation and reflection of the preceding task (Pyka et al., 2009). It is worth noting that during training, more functional connectivities within the DMN changed significantly in the control group than that in the experimental group, while functional connectivity changes within the CEN were only observed in the experimental group (Figure 2). This is similar to the competitive anti-correlation between the DMN and prefrontal regions reported by Fox (Fox et al., 2005), suggesting that during training, the two networks may compete for limited processing resources to serve distinct cognitive processes (Fransson, 2006), and the difference between the two groups may result from the relocation of cerebral resources guided by the true or shame feedback signals (Pyka et al., 2009).

Interactions were found not only within the CEN and the DMN but also between the SN and either of them. In the experimental group, the significantly changed connections were found mainly between the insula and the nodes of the CEN or DMN (Figure 2). Particularly, connections with the right insula were correlated with improvements in behavioral performance (Table 2). In the control group, only the connections between the ACC and DMN were significantly altered, and none of them were correlated with behavioral improvements. Studies that focused on the interactions between the three networks have demonstrated that the insula plays an important role in the mediation between the other two networks. Sridharan et al. proposed that the rFIC plays a major role in switching between the CEN and DMN across task paradigms and stimulus modalities (Sridharan et al., 2008). The rFIC was identified as a causal outflow hub at the junction of the CEN and DMN. Similarly, Menon proposed a triple network model that suggests a causal and potentially critical role of the anterior insula in cognitive control (Menon, 2011). They proposed that one fundamental mechanism underlying such control is a transient signal from the anterior insula, which engages the brain’s attentional, WM and higher-order control processes while disengaging other systems that are not immediately task relevant. Similar to this switching mechanism, the right insula might act as a dispatch center or an integral hub that mediates information flow across other brain networks involved in attentional processing of feedback signals and manipulation of the WM strategy. During training, the activity of lDLPFC was up-regulated by subjects according to the feedback and the insula detected the changes instantly and responded to them correspondingly. The greater the difficulty of the strategies adopted by the subjects, the greater the exchange or communication of information between the SN and other networks. In the experimental group, when the subjects were trying their best to up-regulate the activation of the target ROI with different cognitive strategies, the right insula may have performed its switching role and facilitated task-related information processing by initiating appropriate transient control signals that engaged the CEN while disengaging the DMN (Menon, 2011). The relocation of the limited cognitive resources between the CEN and DMN has been regarded as the support of task performance (Sambataro et al., 2010). The better performance may result from a more optimal allocation done by the right insula, which was less likely to occur in the control group because the subjects received sham feedback and failed to adopt an apposite strategy; thus, they had to address more endogenous stimuli. In other words, although the insula responded to the changes in lDLPFC’s activity, the sham feedback may mislead the subjects to fall flat behavioral performance. Therefore, despite the efforts of subjects in the control group, which were marked by several changes in functional connectivity, it seems that these efforts did not pay off. In addition, it is worth noting that the functional connectivities that were significantly correlated with behavioral improvements fluctuated slightly with obvious up-trends (Figure 3). Previous studies showed that plasticity is an inherent property of the human brain and changes in functional connectivity can take place even after the developmental maturation period (Pascual-Leone et al., 2005; Buschkuehl et al., 2012). Functional connectivity can be enhanced by WM behavioral training (Buschkuehl et al., 2012), and the strength of functional connectivities is modulated by WM load (Fox et al., 2005; Newton et al., 2011). Hence, these slight fluctuations may be caused by the adjustment of a subject’s strategies to one of lower difficulty when he/she finds that he/she is unable to manage the more difficult strategy. Through rtfMRI, the up-trend fluctuations exhibited by these behavior-correlated connectivities, which changed significantly and were associated with the right insula, also demonstrated the training effect on the behaviors and the role of the insula in the training.

In summary, we used ICA and correlation analysis to investigate the interactions between the CEN, DMN and SN during the rtfMRI WM training. The results demonstrated that rtfMRI-based WM training changed the functional connectivities within and between the three networks and among those functional connectivities that were correlated with improvements in behavioral performance, most of them were associated with the right insula. Thus, based on our rtfMRI results, we conclude that the switching role of the insula between the CEN and DMN is also applicable to WM training. This study may not only provide novel insights into the mechanism of WM training, but may also shed light on the clinical treatment and rehabilitation in neurological and psychiatric disorders with the feature of WM impairment.

## Author Contributions

Conceived and designed the experiments: GZ XZ LY. Performed the experiments: GZ. Analyzed the data: QZ. Contributed reagents/materials/analysis tools: QZ XZ LY. Wrote the manuscript: QZ XZ.

## Conflict of Interest Statement

The authors declare that the research was conducted in the absence of any commercial or financial relationships that could be construed as a potential conflict of interest.

## Footnotes

^1^http://www.fil.ion.ucl.ac.uk/spm

^2^http://mialab.mrn.org/software/gift/
